# Neuroendocrine effects of exogenous adropin administration on the hypothalamic pituitary testicular axis in male rats

**DOI:** 10.55730/1300-0144.6164

**Published:** 2025-12-28

**Authors:** Ersen ERASLAN, Ayhan TANYELİ, Mustafa Can GÜLER, Aslı ÖZBEK BİLGİN, Fazile Nur EKİNCİ AKDEMİR, Elif POLAT, Selim ÇOMAKLI, Nezahat KURT, Tuğba BAL TAŞTAN

**Affiliations:** 1 Department of Physiology, Faculty of Medicine, Bandırma Onyedi Eylül University, Bandırma, Turkiye; 2 Department of Physiology, Faculty of Medicine, Atatürk University, Erzurum, Turkiye; 3 Department of Pharmacology, Faculty of Medicine, Erzincan Binali Yıldırım University, Erzincan, Turkiye; 4 Department of Physiology, Erzurum Medicine Faculty, University of Health Sciences, Erzurum, Turkiye; 5 Department of Biochemistry, Faculty of Nutrition and Dietetics, Health Sciences University, Erzurum, Turkiye; 6 Department of Pathology, Faculty of Veterinary Medicine, Atatürk University, Erzurum, Turkiye; 7 Department of Medical Biochemistry, Faculty of Medicine, Erzincan Binali Yıldırım University, Erzincan, Turkiye; 8 Department of Histology and Embryology, Faculty of Medicine, Erzincan Binali Yıldırım University, Erzincan, Turkiye

**Keywords:** Adropin, hypothalamic-pituitary-testicular axis, male infertility, obesity, oxidative stress

## Abstract

**Background/aim:**

Obesity impairs male fertility through metabolic dysfunction, oxidative stress, and disruption of the hypothalamic–pituitary–testicular (HPT) axis. Adropin (ADR), a peptide hormone whose circulating levels are reduced in obesity, plays emerging roles in metabolic homeostasis; however, its involvement in reproductive endocrine regulation remains unclear. The present study was conducted in healthy, nonobese male rats and aimed to investigate the neuroendocrine and testicular effects of exogenous ADR administration, focusing on circulating reproductive hormones, hypothalamic regulatory peptides, and testicular antioxidant pathways.

**Materials and methods:**

Thirty-two male Wistar rats were randomized into control, sham, low-dose ADR (4 μg/kg/day), and high-dose ADR (40 μg/kg/day) groups and treated for 10 days. An enzyme-linked immunosorbent assay (ELISA) was used to measure circulating gonadotropins, testosterone, inhibin B, and activin A. Hypothalamic gonadotropin-releasing hormone (GnRH) and kisspeptin expression, and testicular superoxide dismutase 1 (SOD1) localization were assessed by immunohistochemistry. Brain and testis morphology were examined histologically.

**Results:**

High-dose ADR administration was associated with increased hypothalamic GnRH and kisspeptin expression, accompanied by reduced circulating LH levels, while FSH concentrations remained unchanged. Testosterone and inhibin B levels were higher, whereas activin A levels were lower, in the high-dose ADR group compared with controls. ADR administration was also associated with enhanced testicular SOD1 immunoreactivity and dose-dependent reductions in body weight. No overt histopathological alterations were observed in the cerebral cortex or testicular tissue.

**Conclusion:**

In healthy, nonobese male rats, exogenous ADR administration was associated with changes in central neuroendocrine markers and testicular antioxidant responses without overt histopathological alterations. These findings do not demonstrate improvements in fertility but suggest that ADR may be involved in pathways linking metabolic signals with reproductive endocrine regulation. The potential relevance of these observations to obesity-associated male reproductive dysfunction remains hypothesis-generating and requires confirmation in appropriate disease models and functional reproductive studies.

## Graphical Abstract

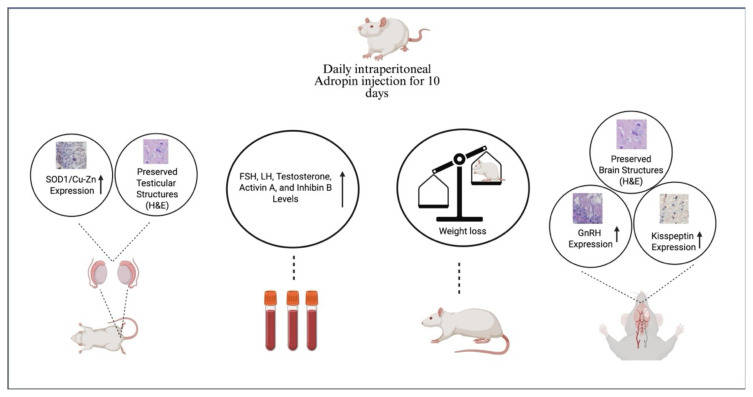

The summary of neuroendocrine effects of exogenous ADR administration on the hypothalamic pituitary testicular axis in male rats. Created with BioRender.com.

## 1. Introduction

Male infertility is a multifactorial condition that arises from a complex interplay between genetic predisposition, endocrine dysfunction, and environmental influences [1]. A substantial body of evidence indicates that obesity is associated with male reproductive dysfunction, largely through metabolic disturbances, chronic low-grade inflammation, oxidative stress, and disruption of the hypothalamic–pituitary–testicular (HPT) axis [2–6]. The present study was conducted in healthy, nonobese male rats. Accordingly, obesity-related mechanisms are discussed here solely to provide a biological context rather than to imply that obesity-associated infertility was directly investigated.

The HPT axis is a tightly regulated neuroendocrine system responsible for maintaining male reproductive homeostasis through coordinated secretion of hypothalamic gonadotropin-releasing hormone (GnRH), luteinizing hormone (LH), follicle-stimulating hormone (FSH), and testosterone [7–10]. GnRH is released in a pulsatile manner from hypothalamic neurons, and this pulsatility is essential for normal gonadotropin secretion and testicular function. Kisspeptin, a hypothalamic neuropeptide encoded by the *Kiss1* gene, is a critical upstream regulator of GnRH neuronal activity and plays a central role in generating and modulating GnRH pulse frequency and amplitude [11–13]. Alterations in kisspeptin signaling have been implicated in hypogonadotropic hypogonadism and reproductive dysfunction in both experimental models and clinical settings.

Because of the pulsatile nature of GnRH and downstream gonadotropin secretion, single-time-point serum hormone measurements provide only a limited snapshot of HPT axis activity and may not fully capture dynamic neuroendocrine regulation. Nonetheless, such measurements remain widely used in experimental studies to assess overall endocrine status, provided that their interpretative limitations are acknowledged.

Adropin (ADR) is a 76-amino-acid peptide hormone encoded by the *Enho* gene, initially identified by Kumar et al., and is emerging as a key player in metabolic regulation [14]. ADR is predominantly expressed in the liver, brain, and endothelial tissues, and exerts diverse effects on glucose metabolism, lipid oxidation, endothelial function, and cardiovascular health [15–17]. Circulating ADR levels are reduced in obesity and metabolic syndrome, suggesting a potential link between metabolic status and ADR signaling [18].

Although ADR has been extensively studied in the context of metabolic regulation, its role in reproductive neuroendocrinology remains incompletely understood. Transcriptomic and immunohistochemical studies indicate that *Enho* expression is present in the central nervous system, including hypothalamic regions involved in neuroendocrine control [19,20]. A putative receptor for ADR, the orphan G-protein-coupled receptor 19 (GPR19), has been proposed based on expression patterns in hypothalamic nuclei and testicular tissue; however, ligand–receptor specificity and downstream signaling mechanisms have not been conclusively established [21]. Therefore, any discussion of ADR signaling pathways should be interpreted with caution, and definitive receptor-mediated mechanisms cannot be assumed.

Given the close integration of metabolic signals with reproductive endocrine regulation, it is plausible that exogenous ADR administration may alter components of the HPT axis via central and/or peripheral pathways. In this context, the present study aimed to examine the neuroendocrine and testicular effects of exogenous ADR administration in healthy male rats by assessing circulating reproductive hormones, hypothalamic GnRH and kisspeptin expression, and testicular antioxidant responses.

## 2. Materials and methods

### 2.1. Chemical agents

**Figure 1:**
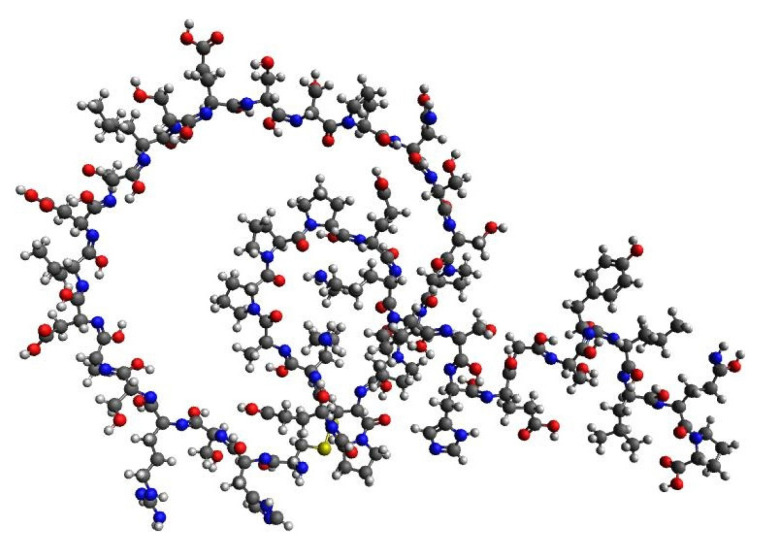
3D molecular structure of ADR. Created with Avogadro version 1.2.0 (http://avogadro.cc/).

ADR (Figure 1) was obtained from Phoenix Pharmaceuticals, Inc. (USA). It was prepared freshly in sterile saline before use. The compound was provided at >95% purity, as verified by the manufacturer via HPLC and mass spectrometry. ADR was stored at −20 °C in its lyophilized form and reconstituted freshly on each experimental day in sterile physiological saline immediately before administration to prevent degradation. All solutions were prepared under sterile conditions, and no preservatives or stabilizers were added.

### 2.2. Ethical approval

The local ethics committee approved all animal procedures for Animal Experiments of Atatürk University (Protocol no. 2015/97). All experimental protocols were conducted in accordance with the Animal Research: Reporting of *In Vivo* Experiments (ARRIVE) guidelines (http://arriveguidelines.org). Every effort was made to minimize animal distress and reduce the number of animals used.

### 2.3. Experimental animals

The animals used in the study were obtained from the Medical Experimental Applications and Research Center (ATADEM) of Atatürk University. Rats were housed in polypropylene cages under a 12:12 h light–dark cycle in a temperature- and humidity-controlled environment. Animals were housed individually throughout the study. No obesity, dietary manipulation, or metabolic disease model was used in this study. All animals were healthy, chow-fed, and maintained under standard laboratory conditions throughout the experimental period. Animals had ad libitum access to standard laboratory chow and water throughout the experiment. Food intake was not quantified, and no pair-feeding or caloric restriction protocol was used. Body weight was recorded daily at the same time of day.

### 2.4. Experimental design

**Figure 2:**
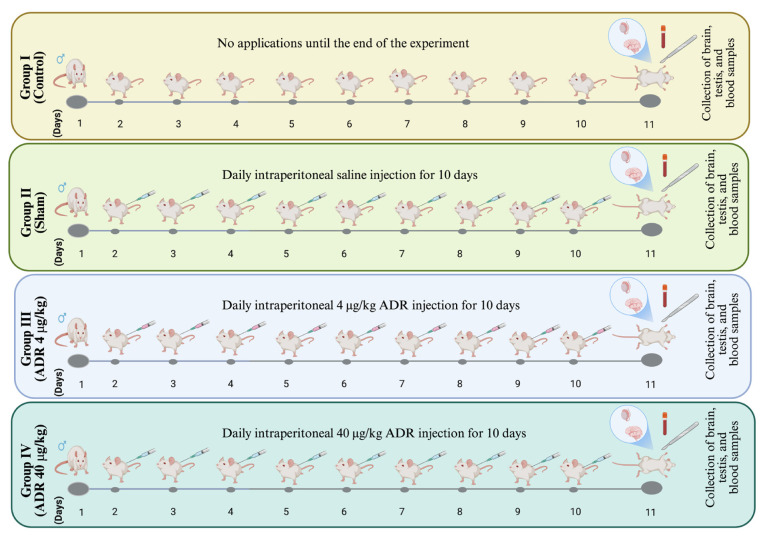
Experimental design and treatment protocol. Thirty-two male Wistar albino rats were randomly allocated to four experimental groups (n = 8 per group) and treated for 10 consecutive days with intraperitoneal injections. The timeline shows daily treatments from day 1 to 10, followed by blood and tissue collection on day 11.

A total of 32 male Wistar albino rats (200–250 g, 12–16 weeks old) were used in this study. The experimental procedure was performed as described in Figure 2. Thirty-two male Wistar albino rats were randomly allocated to four groups (n = 8 per group):

Group I (Control): No applications were performed.
Group II (Sham): Daily intraperitoneal saline for 10 days.
Group III (ADR 4 μg/kg): 4 μg/kg/day ADR was administered intraperitoneally for 10 days.
Group IV (ADR 40 μg/kg): ADR at 40 μg/kg/day was administered intraperitoneally for 10 days.

The selected doses (4 μg/kg and 40 μg/kg) were based on previous studies investigating ADR’s metabolic and hormonal effects in rats [22,23]. In addition, the literature informed our ADR administration duration of 10 days [24].

### 2.5. Tissue and blood collection

On day 11, rats were euthanized by rapid decapitation without prior anesthesia. Anesthetic agents are known to alter HPT axis activity by suppressing GnRH release, modifying LH/testosterone levels, and elevating stress-related hormones [25]. Therefore, anesthesia-free rapid decapitation was preferred to avoid confounding neuroendocrine alterations. Trunk blood was collected within 60 s of cage removal to minimize stress-induced increases in corticosterone and other hormones, affecting the HPA axis [26]. Brain tissue and testes were rapidly harvested and fixed in 10% neutral-buffered formalin for histological and immunohistochemical analyses.

### 2.6. Biochemical analysis

To examine the effects of ADR on gonadotropin levels, an enzyme-linked immunosorbent assay (ELISA; BioTEK Powerwave XS, Winooski, VT, USA) was performed. The measurements were carried out using LH (catalog no. E-EL-R0026), FSH (catalog no. E-EL-R0391), testosterone (catalog no. E-EL-R0389), inhibin B (catalog no. E-EL-R0521), and activin A (catalog no. E-EL-R0001), Elabscience, Beijing, China, commercial ELISA kits in accordance with the manufacturer’s instructions.

All ELISA kits used in this study were validated for use with rat blood samples, ensuring high specificity and minimal cross-reactivity with other analytes. The manufacturer’s datasheets indicate that the assays have been tested across multiple species, including rats, and appropriate validation protocols were followed to ensure reliable hormone measurements.

All serum samples were analyzed in duplicate using commercial ELISA kits according to the manufacturer’s instructions. Four-parameter logistic standard curves were generated for each assay, with coefficients of determination (R^2^) greater than 0.99. No samples were below the lower limit of quantification for LH, FSH, testosterone, inhibin B, or activin A. Therefore, no values required exclusion or imputation. Intraassay coefficients of variation (CVs) reported by the manufacturer were <10% for all ELISA kits used. Intraassay CVs for duplicate measurements in the present study were maintained below 10%; samples exceeding this threshold were reassayed.

### 2.7. Histopathological evaluation

Tissue samples were fixed in 10% neutral buffered formalin for 24–48 h at room temperature, followed by routine dehydration through a graded ethanol series, clearing in xylene, and embedding in paraffin. Paraffin blocks were sectioned at 4–5 μm thickness using a rotary microtome. Sections were mounted on glass slides, deparaffinized, and rehydrated prior to staining. Hematoxylin–eosin (H&E) staining was performed using standard protocols, including nuclear staining with hematoxylin, differentiation, bluing, and cytoplasmic counterstaining with eosin. After dehydration and clearing, sections were coverslipped and examined under light microscopy. A light microscope was used to examine the sections (Olympus BX51, TAPtek camera) for necrosis, hemorrhage, and degeneration.

### 2.8. Immunohistochemical examination

Tissue sections were deparaffinized, rehydrated, and subjected to antigen retrieval using an EDTA solution (pH 8.0) in a boiling water bath. Endogenous peroxidase activity was blocked with 3% hydrogen peroxide, followed by blocking with Ultra V Block. Sections were then incubated overnight at 4 °C with primary antibodies against GnRH (polyclonal; catalog no. sc-8682, 1:100; Santa Cruz Biotechnology), KiSS-1 (monoclonal; catalog no. sc-101246, 1:100; Santa Cruz Biotechnology), or testicular superoxide dismutase 1 (SOD1)/Cu-Zn SOD (polyclonal; catalog no. LS-B9346, 1:100; LSBio).

After primary antibody incubation, sections were treated with a streptavidin–biotin immunoenzymatic secondary antibody system (catalog no. TP-125-HL; Thermo Fisher Scientific), and immunoreactivity was visualized using diaminobenzidine as the chromogen. For each animal, three nonconsecutive sections were analyzed, and five microscopic fields per section were evaluated, resulting in a total of 15 fields per animal for each marker. Fields of interest were selected using standardized anatomical landmarks rather than random sampling. For hypothalamic analyses, regions associated with reproductive neuroendocrine regulation, including the arcuate and anteroventral periventricular nuclei, were consistently examined. For testicular tissue, fields were selected from well-oriented seminiferous tubules and adjacent interstitial areas containing Leydig cells.

Negative controls were included in all immunohistochemical runs by omitting the primary antibody and replacing it with antibody diluent. These sections consistently showed no specific immunostaining, confirming antibody specificity and the absence of nonspecific background staining. All immunohistochemical evaluations and image acquisitions were performed using a light microscope (Olympus BX51) at 400× magnification. Two independent observers blinded to group allocation performed qualitative and quantitative assessments. Any discrepancies were resolved by joint reevaluation to reach a consensus.

Quantitative analysis was performed using ImageJ software. Immunoreactivity was expressed as mean integrated optical density (IOD), calculated as the product of stained area and mean gray value following background subtraction. For each animal, the values from all analyzed fields were averaged to obtain a single representative value per marker.

### 2.9. Statistical analysis

Sample size was estimated a priori using G*Power 3.1 for a one-way ANOVA (fixed effects, omnibus) with four groups, two-sided α = 0.05 and 80% power, assuming a medium effect size (Cohen’s f = 0.25), yielding n = 8 animals per group. For each continuous endpoint, model assumptions were assessed using the Shapiro–Wilk test on residuals (normality) and Levene’s test (homogeneity of variances). One-way ANOVA was used to analyze endpoints with a Bonferroni-adjusted omnibus significance threshold of α = 0.006 (0.05/8).

Tukey’s HSD post hoc comparisons were performed only when the corresponding endpoint met the adjusted omnibus significance threshold. For repeated measurements of body weight, data were analyzed using repeated-measures ANOVA; sphericity was evaluated with Mauchly’s test, and Greenhouse–Geisser corrections were applied when the sphericity assumption was violated.

In addition to p-values, effect size measures were calculated to estimate the magnitude of observed differences. For one-way ANOVA analyses, partial eta squared (η ^2^p) values were reported, with approximate thresholds of 0.01, 0.06, and ≥0.14 indicating small, medium, and large effects, respectively. For selected pairwise comparisons, Cohen’s d was calculated where appropriate.

Data are presented as mean ± SD, and 95% confidence intervals (95% CI) were provided for key hormonal outcomes. For immunohistochemical analyses, quantitative outcomes were derived from integrated optical density measurements in ImageJ. All statistical tests were two-sided. Statistical analyses were performed using SPSS software (version 22.0), and graphical representations were generated using GraphPad Prism (version 8.0).

## 3. Results

### 3.1. Biochemical parameters

**Figure 3:**
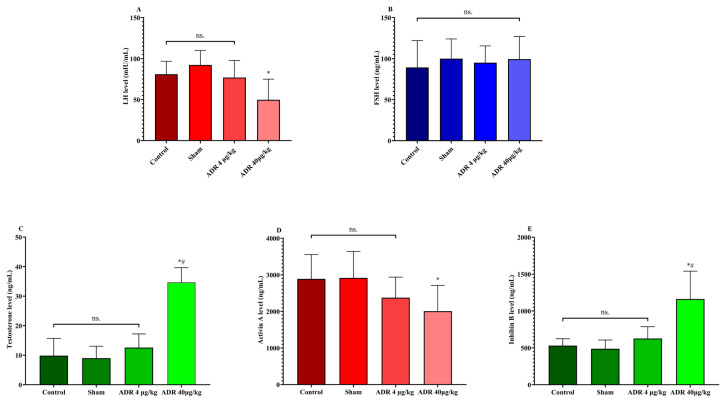
Dose-dependent effects of ADR administration on circulating reproductive hormones: A) high-dose ADR significantly decreased LH versus control/sham, B) FSH levels remained unchanged across all groups, C) testosterone concentrations were significantly elevated following high-dose ADR treatment, D) activin A levels demonstrated a significant reduction in the high-dose ADR group, E) inhibin B levels significantly increased in the high-dose ADR group. Data are presented as mean ± SD with individual data points (n = 8 per group). One-way ANOVA with Bonferroni-adjusted omnibus α = 0.006 (0.05/8); Tukey’s HSD pairwise comparisons are reported only when the corresponding omnibus test met this adjusted threshold. *p < 0.001 vs. control; #p < 0.001 vs. sham. “ns” over a bracket denotes a nonsignificant Tukey comparison (p ≥ 0.05). Horizontal lines above bars indicate significant pairwise comparisons.

The effects of 10-day ADR administration on circulating reproductive hormones are presented in Figure 3 and Table 1. Circulating LH levels were lower in the high-dose ADR group compared with the control and sham groups (p < 0.001), whereas no significant difference was observed between the low-dose ADR and control groups (Figure 3A). FSH concentrations did not differ significantly among the experimental groups (Figure 3B).

Testosterone levels were higher in the high-dose ADR group than in the control and sham groups (p < 0.001), while the low-dose group showed intermediate levels (Figure 3C). Activin A concentrations were lower in the high-dose ADR group compared with the control and sham groups (p < 0.001) (Figure 3D). In contrast, inhibin B levels were higher in the high-dose ADR group compared with the control and sham groups (p < 0.001) (Figure 3E).

### 3.2. Cerebral cortex histology

**Figure 4:**
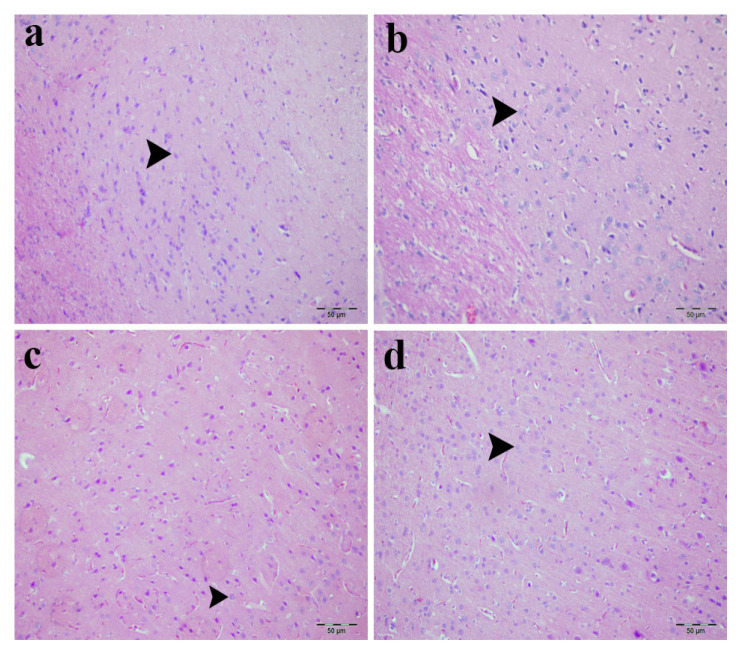
Histopathological examination of cerebral cortex tissue architecture. Representative photomicrographs of H&E-stained brain sections: a) control group showed normal neuronal morphology with intact nuclei and preserved cytoplasmic architecture, b) sham group displayed comparable cortical structure with well-preserved neurons, c) low-dose ADR group (4 μg/kg) preserved intact cortical organization and preserved neuronal morphology, d) high-dose ADR group exhibited maintained neuronal integrity with normal nuclear and cytoplasmic features. Arrowheads indicate representative neurons with preserved morphology. Original magnification: 400×; scale bar: 50 μm.

Histopathological examination of cerebral cortex sections revealed no gross cortical abnormalities or evidence of necrosis or inflammation in any group (Figure 4). Control and sham groups exhibited normal neuronal morphology with intact nuclei, nucleoli, and homogeneous cytoplasm (Figures 4a–4b). In the ADR-treated groups, cortical organization remained comparable to controls, with neurons essentially showing regular nuclear and cytoplasmic features (Figures 4c–4d). No necrosis, hemorrhage, or inflammatory infiltration was detected in any group.

### 3.3. Testicular histology

**Figure 5:**
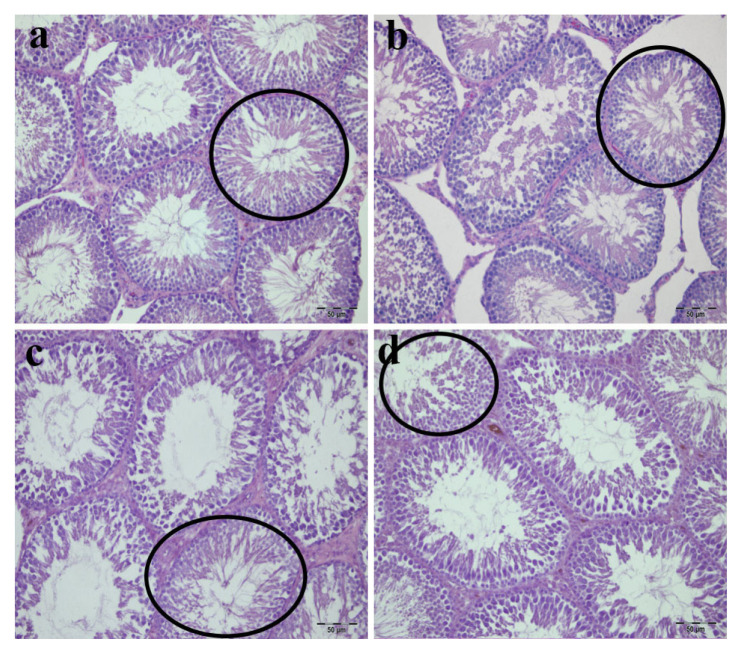
Testicular histoarchitecture. Representative photomicrographs of H&E-stained testis sections: a) control group displaying well-preserved seminiferous tubule organization and normal tissue morphology, b) sham group showed comparable tubular structure and intact testicular architecture, c) low-dose ADR group demonstrated preserved germinal epithelium thickness and overall normal tissue structure, d) high-dose ADR group also exhibited intact testicular architecture with maintained tubular organization. Circles highlight seminiferous tubules with preserved morphology. Leydig cells in the interstitial spaces appeared morphologically normal in all groups. Original magnification: 400×; scale bar: 50 μm.

Testicular histology demonstrated regular tissue architecture in all groups (Figure 5). Control and sham groups displayed organized seminiferous tubules with normal germinal epithelium and intact interstitial structure (Figures 5a–5b). Similarly, low- and high-dose ADR groups maintained tubular organization and preserved germinal epithelium thickness (Figure 5c–d). Leydig cells showed comparable morphology across all groups.

### 3.4. Hypothalamic GnRH immunoreactivity

**Figure 6:**
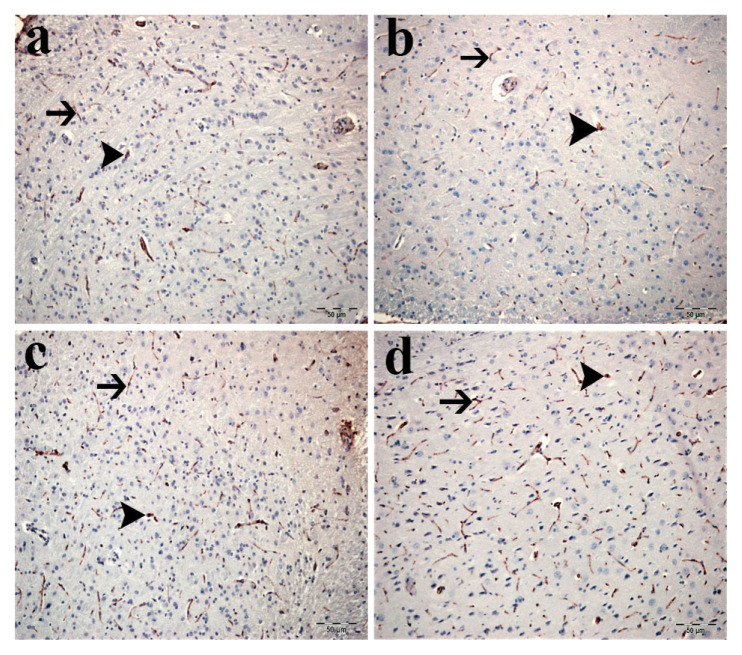
Hypothalamic GnRH expression analysis. Representative immunohistochemical staining for GnRH in brain tissue: a) control group showing limited GnRH-immunopositive neurons with weak cytoplasmic staining intensity, b) sham group displaying similar low-level GnRH expression pattern as controls, c) low-dose ADR group demonstrating noticeable increase in both number and staining intensity of GnRH-positive hypothalamic neurons, d) high-dose ADR group exhibiting marked upregulation with dense, widespread cytoplasmic immunoreactivity. Arrowheads indicate GnRH-immunopositive neuronal cell bodies; arrows denote cytoplasmic immunostaining. Original magnification: 400×; scale bar: 50 μm.

Immunohistochemical analysis demonstrated differences in GnRH immunoreactivity among groups (Figure 6, Table 2). Control and sham groups displayed baseline GnRH immunoreactivity with sparse positive neurons showing weak-to-moderate cytoplasmic staining (Figures 6a–6b). Increased GnRH immunoreactivity was observed in the low-dose ADR group (Figure 6c), with further increases in the high-dose ADR group (Figure 6d).

### 3.5. Hypothalamic kisspeptin expression

**Figure 7:**
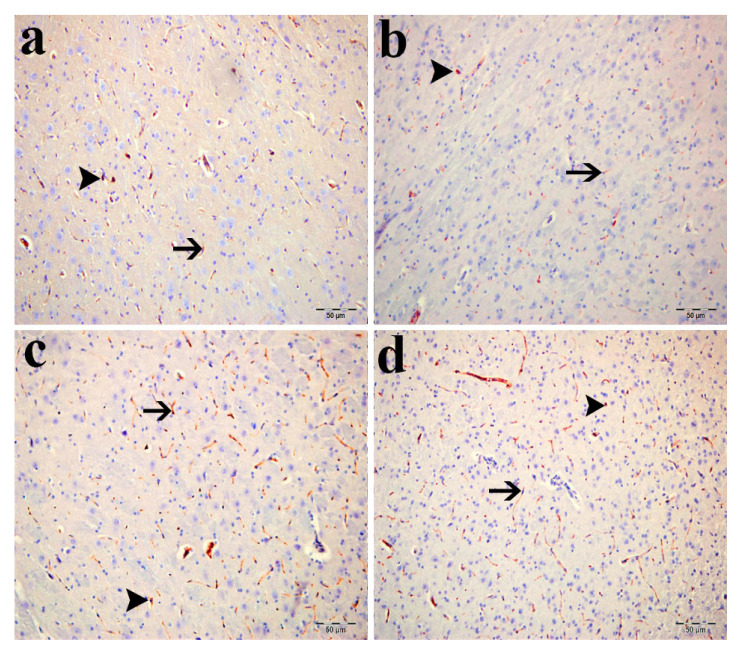
Kisspeptin expression in hypothalamic reproductive control centers. Representative immunohistochemical localization of kisspeptin in brain sections: a) control group exhibiting sparse, weakly immunoreactive kisspeptin-positive neurons, b) sham group showing similar low-level kisspeptin expression, c) low-dose ADR group displaying increased kisspeptin immunopositivity in regions associated with reproductive neuroendocrine control, d) high-dose ADR group demonstrating maximal kisspeptin expression with abundant, intensely stained neuronal populations. Arrowheads and arrows identify kisspeptin-immunopositive neurons. Original magnification: 400×; scale bar: 50 μm.

Kisspeptin immunoreactivity differed across experimental groups (Figure 7, Table 2). Minimal immunopositivity was observed in the control and sham groups (Figures 7a–7b). The low-dose ADR group exhibited higher kisspeptin immunoreactivity than controls (Figure 7c), while the highest immunopositivity scores were observed in the high-dose ADR group (Figure 7d).

### 3.6. Testicular SOD1 expression

**Figure 8:**
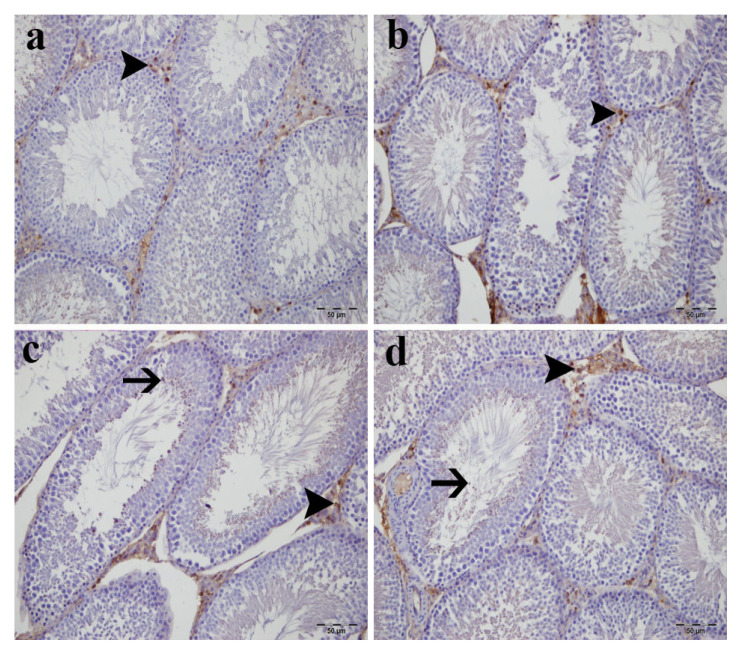
Testicular antioxidant enzyme expression and cellular localization. Representative SOD1/Cu-Zn immunohistochemistry in testicular tissue: a) control group showing limited SOD1 immunoreactivity primarily in basal germ cells and scattered interstitial areas, b) sham group displaying similar weak cytoplasmic staining pattern, c) Low-dose ADR group exhibiting moderate increases in SOD1 expression within spermatocytes and Leydig cell populations, d) high-dose ADR group demonstrating marked SOD1 immunoreactivity throughout seminiferous epithelium and prominent staining in interstitial Leydig cells, indicating enhanced antioxidant response. Brown coloration represents positive immunostaining. Arrowheads indicate immunopositive Leydig cells; arrows denote positive spermatocytes. Original magnification: 400×; scale bar: 50 μm.

SOD1 immunoreactivity in testicular tissue varied among groups (Figure 8, Table 2). Control and sham groups exhibited minimal SOD1 immunoreactivity, primarily localized to basal spermatogonia and scattered Leydig cells (Figures 8a–8b). Increased SOD1 immunoreactivity was observed in the low-dose ADR group (Figure 8c), with the highest levels detected in the high-dose ADR group (Figure 8d).

### 3.7. Body weight dynamics

Changes in body weight during the experimental period are summarized in Table 3. Control and sham groups showed modest weight gain over the 10 days. In contrast, animals receiving ADR exhibited reductions in body weight, with greater reductions observed in the high-dose group. Body weight data are reported descriptively, and no causal relationship between weight changes and hormonal alterations was inferred.

## 4. Discussion

This study provides new evidence that exogenous ADR administration is associated with alterations in multiple components of the HPT axis in male rats, including changes in central neuroendocrine mediators and testicular antioxidant pathways. Increases in testosterone and inhibin B, together with reductions in LH and activin A, occurred alongside enhanced hypothalamic GnRH and kisspeptin immunoreactivity, without gross histological disruption in cerebral or testicular tissues. These findings suggest that ADR may influence both central and peripheral aspects of reproductive endocrinology; however, their interpretation requires careful consideration of regulatory complexity and experimental limitations.

The HPT axis is a tightly regulated neuroendocrine system integrating metabolic, hormonal, and environmental cues to maintain male reproductive homeostasis [7,27]. Among central regulators, kisspeptin plays a pivotal role in GnRH pulsatility and subsequent LH/FSH secretion [28], with disruptions in kisspeptin signaling contributing to hypogonadotropic hypogonadism [13]. In the present study, ADR treatment was associated with increased immunoreactivity for GnRH and kisspeptin in key hypothalamic nuclei. However, immunohistochemical changes reflect peptide expression or availability and do not directly capture pulse frequency or amplitude, which are critical determinants of downstream gonadotropin release.

An important observation is the apparent discrepancy between increased GnRH/kisspeptin expression and reduced circulating LH levels. Several physiological and methodological factors may account for this pattern. First, alterations in GnRH or kisspeptin expression do not necessarily translate into increased LH secretion if pulse characteristics are altered. Second, the reduction in LH occurred in parallel with elevated testosterone concentrations. Testosterone exerts well-established negative feedback at both hypothalamic and pituitary levels, which may suppress LH secretion independently of upstream GnRH neuronal activity. Third, the present study relied on a single terminal time point for hormone measurements. Given the dynamic and pulsatile nature of the HPT axis, single measurements provide only a snapshot of endocrine status and may not reflect transient or phase-dependent changes in gonadotropin secretion. Together, these considerations indicate that the GnRH/kisspeptin–LH dissociation should be interpreted cautiously.

Although the orphan GPR19 has been proposed as a putative ADR receptor, its ligand specificity remains controversial and incompletely defined [21]. Therefore, while enhanced kisspeptin and GnRH expression is consistent with ADR-related neuronal modulation, definitive conclusions regarding receptor-mediated mechanisms cannot be drawn. ADR has been shown to influence neuronal excitability within hypothalamic regions such as the paraventricular nucleus [29], suggesting that indirect central pathways may contribute to HPT axis regulation. Future studies employing receptor-specific blockade, knockdown, or cell-targeted genetic approaches will be necessary to clarify ADR’s precise mode of action.

A particularly intriguing finding is the concurrent increase in inhibin B and kisspeptin expression, together with reduced activin A levels. Activin–inhibin signaling is known to influence *Kiss1* gene regulation, with activin A and inhibin A reported to enhance hypothalamic *Kiss1* expression, whereas inhibin B has been associated with suppressive effects [30]. The pattern observed here, therefore, appears paradoxical. Several nonmutually exclusive explanations may be considered, including temporal dissociation between circulating hormones and central gene regulation, compensatory feedback responses within the HPT axis, and species- or sex-specific regulatory mechanisms. Importantly, the single-time-point design and absence of gene-expression or receptor-signaling analyses limit mechanistic inference, and longitudinal or cell-specific studies will be required to clarify these interactions.

Weight loss represents a major confounding factor in the interpretation of the present findings. ADR-treated animals exhibited dose-dependent reductions in body weight, consistent with previous reports demonstrating ADR’s role in insulin sensitivity, lipid oxidation, and feeding behavior [14–18]. Because metabolic status strongly influences reproductive hormone profiles, it is not possible to distinguish direct endocrine effects of ADR from secondary effects mediated by weight loss or altered energy balance. Improvements in testosterone and inhibin B may therefore partially reflect metabolic changes rather than ADR-specific actions. The absence of pair-fed or weight-matched controls is a critical limitation, and future studies should incorporate such designs to disentangle weight-dependent and weight-independent mechanisms.

Oxidative stress is a key link between metabolic dysfunction and impaired male fertility. Excess reactive oxygen species disrupt spermatogenesis, damage sperm DNA, impair Leydig cell steroidogenesis, and reduce sperm quality [31,32]. SOD1 is a key antioxidant enzyme involved in the detoxification of superoxide radicals, and its upregulation suggests enhanced testicular antioxidant defenses [31]. In this study, ADR was associated with increased testicular SOD1 immunoreactivity, particularly in spermatocytes and Leydig cells. While this suggests enhanced antioxidant defenses, functional reproductive parameters were not assessed. Sperm count, motility, morphology, and DNA integrity were not measured, and therefore, no conclusions regarding improved fertility can be drawn. Functional implications of ADR-associated antioxidant changes thus remain speculative.

Histological preservation of cortical and testicular architecture across groups is reassuring but should not be interpreted as evidence of tissue safety. Routine H&E staining cannot detect subtle neural or germ-cell toxicity, apoptotic signaling, or ultrastructural injury. More sensitive analyses will be required to comprehensively evaluate tissue-level effects of ADR.

Taken together, the present findings support the hypothesis that ADR is involved in integrating metabolic and neuroendocrine signals within the male reproductive axis. However, translational and therapeutic implications should be considered preliminary. Substantial mechanistic uncertainties remain, including receptor identity, the paradoxical inhibin–kisspeptin pattern, and the confounding influence of weight loss. Addressing these issues through mechanistic, longitudinal, and functional reproductive studies will be essential before ADR-based interventions can be considered in clinical contexts.

The ADR doses used in the present study (4 and 40 μg/kg/day) were selected based on previous rodent studies reporting metabolic and neuroendocrine effects of exogenous ADR administration [22,23]. However, it remains unclear whether these doses correspond to physiological, near-physiological, or supraphysiological exposure levels relative to endogenous circulating ADR in humans. At present, limited pharmacokinetic data are available regarding ADR bioavailability, tissue distribution, and dose–exposure relationships across species. Therefore, the translational relevance of the dosing regimen used here should be interpreted with caution.

Several limitations of the present study should be acknowledged. First, the study was conducted in healthy, nonobese male rats, and therefore, the findings cannot be directly extrapolated to obesity-associated reproductive dysfunction. Second, ADR-associated weight loss represents a major potential confounder, and in the absence of pair-fed or weight-matched control groups, direct endocrine effects of ADR cannot be clearly distinguished from secondary effects mediated by changes in body weight or energy balance. Third, the assessment of reproductive hormones was based on single terminal time-point measurements, which do not capture the pulsatile nature of GnRH and gonadotropin secretion. Fourth, functional reproductive outcomes, including sperm count, motility, morphology, and fertility indices, were not evaluated, precluding conclusions regarding reproductive competence. Finally, the study provides limited mechanistic molecular insight, as gene-expression analyses, receptor-specific signaling assays, and pathway-level investigations were not performed. These limitations should be considered when interpreting the findings, which should be viewed as hypothesis-generating and informative for the design of future mechanistic and translational studies.

## Figures and Tables

**Table 1:** Reproductive hormone concentrations following ADR treatment.

Hormone	Control	Sham	Low-dose ADR	High-dose ADR	p-value†
**FSH (mIU/mL)**	5.5 ± 0.7 (95% CI: 4.9–6.1)	5.4 ± 0.6 (95% CI: 4.9–5.9)	5.3 ± 0.8 (95% CI: 4.6–6.0)	5.2 ± 0.8 (95% CI: 4.5–5.9)	0.766
**LH (mIU/mL)**	4.2 ± 0.5 (95% CI: 3.8–4.6)	4.1 ± 0.4 (95% CI: 3.8–4.4)	3.5 ± 0.5 (95% CI: 3.1–3.9)	2.8 ± 0.4 (95% CI: 2.5–3.1)**	<0.001
**Testosterone (ng/mL)**	32.1 ± 4.8 (95% CI: 28.1–36.1)	33.2 ± 5.1 (95% CI: 28.9–37.5)	37.8 ± 4.9 (95% CI: 33.7–41.9)	45.3 ± 5.2 (95% CI: 41.0–49.6)**	<0.001
**Activin A (pg/mL)**	68.9 ± 7.8 (95% CI: 62.4–75.4)	67.5 ± 7.2 (95% CI: 61.5–73.5)	56.8 ± 6.9 (95% CI: 51.0–62.6)	45.2 ± 6.3 (95% CI: 40.0–50.4)**	<0.001
**Inhibin B (pg/mL)**	198.4 ± 22.1 (95% CI: 180.0–216.8)	201.3 ± 24.5 (95% CI: 181.0–221.6)	238.7 ± 25.6 (95% CI: 217.4–260.0)	285.6 ± 28.3 (95% CI: 262.1–309.1)**	<0.001

Values are mean ± SD (n = 8/group), with 95% confidence intervals (CIs) shown in parentheses.

† Omnibus one-way ANOVA p-value (Bonferroni-adjusted α = 0.006 across endpoints).

** indicates Tukey’s HSD vs. control and sham at p < 0.001.

**Table 2:** ImageJ analysis results of GnRH, Kisspeptin, and SOD1/Cu-Zn.

Group	GnRH immunopositivity	Kisspeptin immunopositivity	SOD1/Cu-Zn immunopositivity
**Control**	1.07 ± 0.05	0.62 ± 0.06	0.70 ± 0.14
**Sham**	1.35 ± 0.07	0.43 ± 0.04	1.16 ± 0.20
**Low-dose ADR**	1.90 ± 0.08*	1.63 ± 0.05**	4.52 ± 0.13**
**High-dose ADR**	2.55 ± 0.08**	1.81 ± 0.06**	4.90 ± 0.13**

Immunopositivity scores represent the mean IOD values obtained using ImageJ. IOD was calculated as the product of the positively stained area and the mean gray value after background subtraction. Higher values indicate greater immunoreactivity. For each animal, values were averaged across all analyzed fields to generate a single representative score.

* Omnibus one-way ANOVA at Bonferroni-adjusted α = 0.006 across endpoints. Pairwise significance versus control is from Tukey’s HSD (

* p < 0.05;

** p < 0.001).

**Table 3:** Daily weight changes among the experimental groups.

Group	Initial weight (g)	Final weight (g)	Weight change (g)	% Change
**Control**	250 ± 5.6	255 ± 5.2	+5 ± 1.4	+2.0%
**Sham**	251 ± 6.1	256 ± 5.8	+5 ± 1.3	+2.0%
**Low-dose ADR**	252 ± 5.9	248 ± 5.5	−4 ± 1.2	−1.6%
**High-dose ADR**	253 ± 6.0	245.5 ± 5.7	−7.5 ± 1.2**	−3.0%

Values are mean ± SD (n = 8/group).

** p < 0.001 vs. control and sham (repeated-measures ANOVA with Bonferroni-adjusted pairwise comparisons).
